# Are vent crab behavioral preferences adaptations for habitat choice?

**DOI:** 10.1371/journal.pone.0182649

**Published:** 2017-09-07

**Authors:** Hans-Uwe Dahms, Li-Chun Tseng, Jiang-Shiou Hwang

**Affiliations:** 1 Department of Biomedical Science and Environmental Biology, Kaohsiung Medical University, Kaohsiung, Taiwan; 2 Department of Marine Biotechnology and Resources, National Sun Yat-sen University, Kaohsiung, Taiwan; 3 Institute of Marine Biology, National Taiwan Ocean University, Keelung, Taiwan; Zhejiang University College of Life Sciences, CHINA

## Abstract

Hydrothermal vent organisms are adapted to their extreme and patchily distributed habitats. They are expected to have evolved mechanisms that keep them in their specific habitation. Since little is known about the recruitment or habitat selection of HV organisms such as brachyurans, we examined the properties of several hydrothermal vent-associated cues on the behavior of the hydrothermal vent (HV) crab *Xenograpsus testudinatus* in the laboratory that were contrasted by the offering of non-vent cues. This crab species is endemic and dominates the vent fauna of Turtle Island off the NE coast of Taiwan. HV crabs were separately and in combination offered the following vent-specific cues: (1) sulfuric sediment, (3) air-bubbling, (4) elevated temperature, (5) dead settled zooplankton, (7) other crabs, and (8) shade. The non-vent-specific cues were: (2) quarz sediment, (6) dead fish, (8) light. These cues were provided on either side of a two-choice chamber. The movement of individual crabs was monitored: as initial and final choices, and as the proportion of time the crabs spent in each compartment (resident time). Cues were offered alone and no such cue as a control in the same set-up. Sulfuric sediments and dead fish were significantly more attractive to females, and other crabs irrespective of gender were significantly more attractive to males. When compared to expected distributions, crabs, irrespective of gender, significantly avoided light and tended to select other crabs, air-bubbling, sulfuric sediment, elevated temperature, dead fish, dead zooplankton, and quarz sediments in the order of decreasing importance. Data do not support the hypothesis that dead settled zooplankton was particularly attractive nor that the other gender was selected. A combination of several vent-associated cues (sulfuric sediment, elevated temperature, air-bubbling) facilitated the strongest attraction to the crabs as reflected by all response variables. The ‘first choice’ responses were always consistent with the side of the choice compartment in which they spent the longest amount of time (resident time), but not with the ‘final choice’ of crabs, suggesting that the ‘resident time’ in addition to their ‘first choice’ is more reliable than just the ‘final choice’. The present results provide the first indication that several vent-associated habitat cues function as attractors for HV crabs. Habitat choice is also reflected by crab larval distribution in the field which tend to stay near the bottom not to be carried away from their specific habitat.

## Introduction

Hydrothermal vents (HVs) are extreme habitats with unique physicochemical and geological conditions [1]. They are most common but not confined to the deep-sea [2]. HVs show variable and extreme conditions of physicochemical parameters, such as high temperature, high sulfide and metal content, high level of carbon dioxide, low level of oxygen and low pH [3]. Hydrothermal vents also release a great amount of chemical constituents into the marine environment, elevating the concentrations of chemical compounds around the environment [4]. This concentration is higher by one to two orders of magnitude than that in mussels collected from heavily polluted marine environments, such as Minamata Bay [5]. The hydrothermal vents around Kueishan Island, also called Turtle Island. This is located in northeastern Taiwan near the southern end of the Okinawa Trough, release hydrothermal fluids which contain higher concentrations of major and trace elements, pure sulfur and extremely acidic thermal fluids, with pH values as low as 1.52 [6]. This environment is naturally enriched in terms of trace metals, and thus provide a suitable template for a naturally “polluted site” to compare with environments that are affected by anthropogenic pollution [7].

Biological communities associated with HVs show behavioral, physiological, morphological, and reproductive adaptations [8–9]. This holds for symbiotic associations [10], physiological and biochemical systems for sulfide detoxification [11], and behavioral and molecular responses to high temperature [12], and specialized sensory organs to locate hot chimneys [13]. HV habitats are characterized by the spatially and temporally variable input of hydrothermal fluids [14]. Whereas the particular vent fauna seems to be adapted to their HV habitat, other organisms being translocated there are at times lethally affected [15]. The marine environment adjacent to Kueishan Island provides a natural laboratory for different studies [16]. We examined the attractive properties of several vent-associated cues on the vent crab *Xenograpsus testudinatus* [17] in the laboratory in a two-choice set-up. This crab is the only metazoan close to the vents off Turtle Island.

Turtle Island is a volcanic island originating from the Holocene close offshore at the NE coast of Taiwan [18]. The HVs of Turtle Island are located at a tectonic junction of the fault system extension of Taiwan and the southern rifting end of the Okinawa Trough only 60 miles south of those from the Okinawa Trough [19]. A cluster of more than 50 HVs, detectable by side scan sonar and echo sounder sensors, at water depths between 10 m and 80 m off the southeastern tip of Turtle Island, emits hydrothermal fluids and volcanic gases [20]. Sulfur chimneys, formed by the metabolic activity of sulfur bacteria [21], and the condensation of the sulfur contained in hydrothermal fluids, can usually be seen around such HVs at the chimney outlets worldwide [22] as well as at Turtle Island [19]. The hydrothermal vent discharges are acidic, hot, and sulphur-rich [15]. The gases show a similar composition of low temperature fumaroles, with low SO_2_ and HCl but high C0_2_ and H_2_S contents of a mantle source region without significant crust contamination [23]. There are only a few macrofaunal species found at this particular vent site. The fauna is dominated by HV crabs [24]. McClay [25] summarized in a review of hydrothermal vents that many new species of unusual crabs were found. In a review of HV decapods wordwild, Martin and Haney [26] listed 125 crab species belonging to 33 families. We studied *Xenograpsus testudinatus* Ng et al. 2000 [17] described in a volume edited by Hwang and co-workers in 1998 [18]. *X*. *testudinatus* was discovered as the third species of the genus *Xenograpsus* Takeda and Kurata, 1977 (Bythograeidae) from shallow water hydrothermal vents off the east coast of Taiwan [27]. Meanwhile, several investigations were carried out with these crabs, such as a study of their mitogenome [28]. Large numbers of these HV crabs are hiding in the pits, fissures, and crevices of the sulfur chimneys can be observed. It is estimated that the population density of the crab can be up to 287 individuals per square meter in waters ranging from 12m to 30m depth (pers. unpubl. data). This habitat seems to provide sufficient food for crabs and is otherwise free of predators.

Representatives of *Xenograpsus* were found in all the known hydrothermal vents—in the western Pacific Ocean [29]. Information published on these brachyurans include studies on their biogeography and evolution [30–31]. Crabs have been studied at several HV sites for their reproductive biology [32] and ecology and distribution [33], and the behaviour of their larval stages [34]. But little is known about the recruitment or habitat selection of HV crabs.

The objective of the present study is to evaluate aspects of the supply side ecology of HV crabs. Specifically, we hypothesize that there will be adaptations towards larval retainment and cues for home finding in crabs. Hence, we studied: (1) the spatiotemporal field distribution of *X*. *testudinatus* larvae in the shallow HVs of Turtle Island (2) adult behavior in two-choice experiments designed to test towards different HV-associated single cues or a combination of cues (3).

## Material and methods

The Ministry of Science and Technology of Taiwan, ROC, granted permission. Medicine, Kaohsiung Medical University (KMU), the Asia-Pacific Ocean Research Center of the Department of Oceanography (No. 76211194) in the frame of the KMU/NSYSU cooperation, and MOST 105-2621-M-037-001 to T.H. Shih.

### Crab larva collection

**Fig 1:**
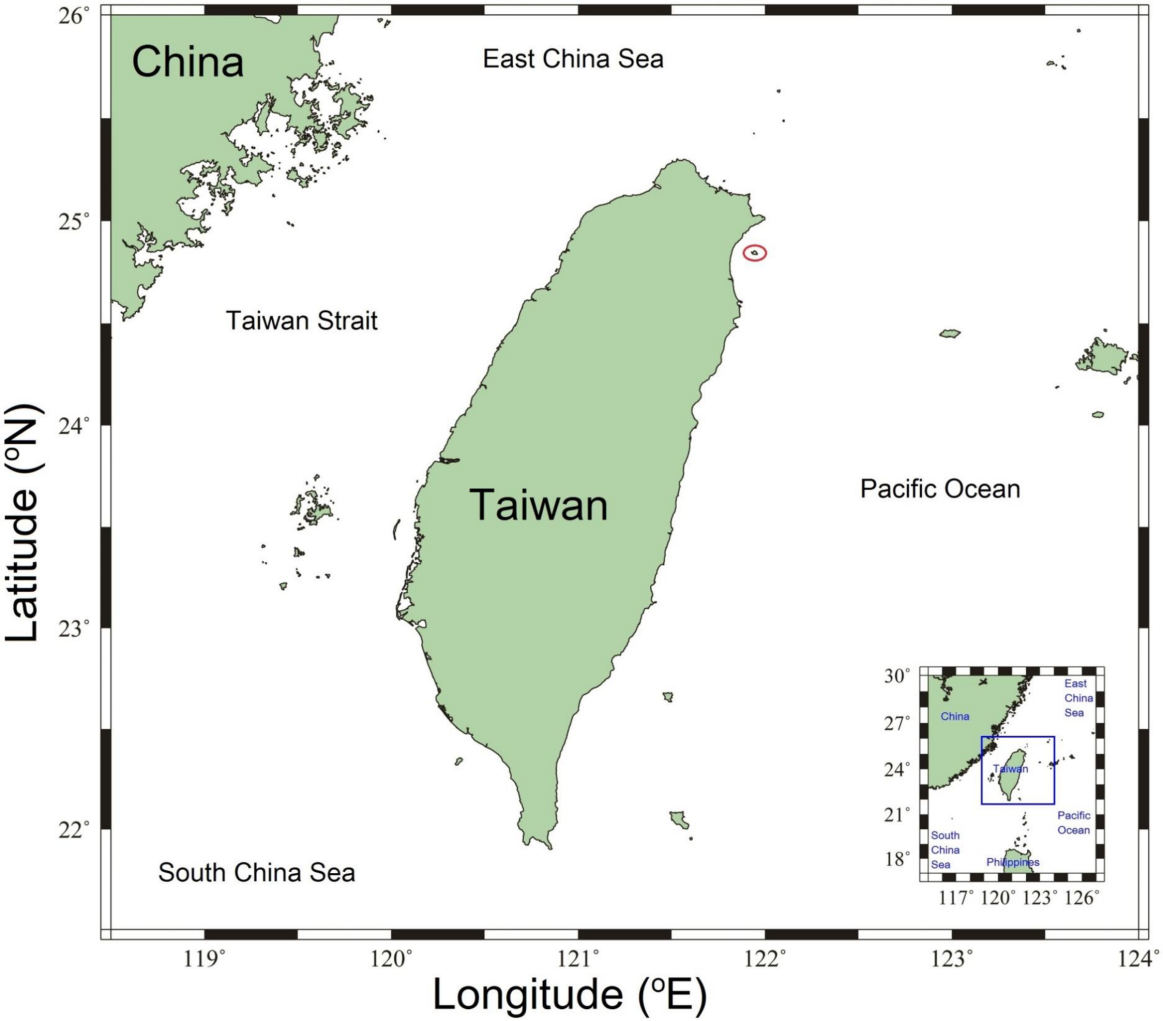
Study site at Turtle Island (Kueishan Tao), red spot represents the location of the hydrothermal vent site.

Meroplanktonic crab larvae were collected by oblique tows with a standard North Pacific zooplankton net (mouth diameter 45 cm and a mesh size of 333 μm) from surface waters (0-1m depth), midwater (6m depth), and above ground (12-15m depth) at an experimental vent site A of Turtle Island in 4 replicates (Fig 1). A Hydrobios (Kiel, Germany) flowmeter was strapped in the center of the net opening for later estimation of the seawater volume filtered. The plankton samples were fixed in 4% buffered formalin. Meroplanktonic larvae were later identified and counted in the laboratory.

### Adult crab collection

Adults of the vent crab *X*. *testudinatus* were collected for behavioral experimentation from the vent site of Turtle Island by SCUBA dives at a depth of about 18m. The crabs were rapidly transferred to aquaria filled with aerated surface seawater from the vent site as soon as they reached the Research Vessel. We also provided air-supply during the rapid and cautious transport of the crabs to the laboratory.

### Binary-choice behavioral experiment

**Fig 2:**
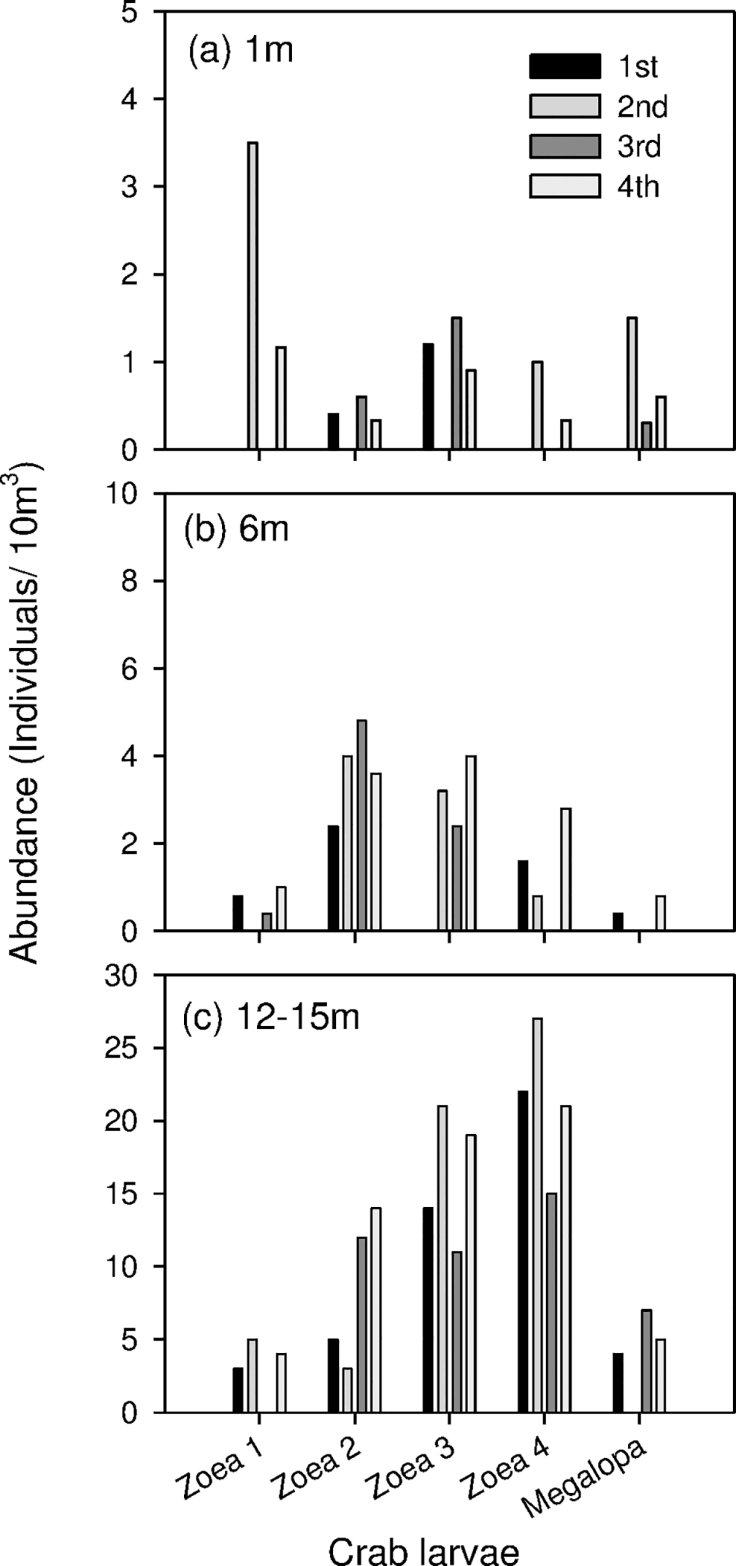
**Abundance variations of 4 replicate collections for crab larvae distribution in three depths:** 0–1 m (a), 6 m (b) and 12–15 m (c).

A binary-choice tank (95 x 60 x 45 cm) (Fig 2) was used to investigate the responses of both gender of *X*. *testudinatus* to different cues in a 2-choice experiment.

Individual crabs were introduced in the middle of the chamber in a round untransparent beaker with an opening at the inverted bottom. The beaker was manually lifted for each individual run of the experiment. The crabs then could choose to move to either the left or the right side where alternating different materials were deposited behind a glass wall that allowed water communication through a gap at the bottom. This gab also allowed physical (warmer or colder water) or physical (chemical taste) [35] cues to escape and crabs to sense such cues. Uniform ambient lightning was provided by white fluorescent ceiling illumination tubes of the wetlab at the National Taiwan Ocean University (NTOU) with a similar set-up as described by Dahms et al. [36] for the maintenance of the crabs. The temperature in the tank was maintained at 24 ± 1°C at all times by a laboratory air conditioner. No aeration was provided during experimentation. In total were 8 items subjected for the behavioral choice of hydrothermal vent crabs. Table 1 provided the details of each experimental item.

**Table 1:** Test results and *p* values of binary-choice for female and male crabs. The letter in the parentheses was the prefered choice of crab to the test site (T) and control site (C), equal mean choice to test site and control site was 50%:50%.

Test item	Female		Male	
	Initial	Final	Initial	Final
Sulf sed	(T) 0.001	(T) 0.005	(T) 0.011	(T) 0.005
Quartz sed	(T)	(T)	(T)	(equal)
Elevated temp	(T)	(T)	(T)	(T)
Low temp	(C)	(C)	(C)	(C)
Zoopl dead	(T)	(T)	(T) 0.048	(T)
Fish dead	(T) 0.005	(T) 0.048	(T) 0.024	(T)
Light	(C)	(C) 0.048	(C)	(C) 0.011
Shade	(T)	(equal)	(equal)	(T)
Male	(T)	(T) 0.048	(T)	(T)
Female	(T) 0.048	(T)	(T) 0.048	(T) 0.048
Group of 6	(T) 0.024	(T) 0.011	(T) <0.001	(T) 0.001
Air bubbling	(T) 0.002	(T) 0.005	(T) 0.011	(T) 0.002
Mix factors I	(T) <0.001	(T) <0.001	(T) <0.001	(T) <0.001
Mix factors II	(C)	(C)	(C)	(C)

T–treatment group; C–control group

(1) Sulfuric sediment of 0.8 mm mean grain size was collected from around the chimney of the vent site, sieved through a 0.9 and 0.7 mm geological sieve and kept airtight in plastic bags in the refrigerator until experimentation. On the treatment side 10g of sulfuric sediment was added prior to the choice test.

(2) Quartz sediment of 0.8 mm grain size was obtained from a commercial aquarium supplyer and kept dry until experimentation. On the treatment side 10g were added prior to the choice test.

(4) Elevated/ low temperature. Elevated temperature was provided by a 100W commercial aquarium heater that provided a gradient of 10°C from the treatment compartment (35°C) to the main test arena (25°C). Low temperature provided a similar gradient of 10°C by adding seawater ice cubes to the treatment compartment.

(5) Dead settled zooplankton (5 g) was obtained alive from off Turtle Island waters, frozen, and defrosted prior to experimentation.

(6) Muscle meat of mackerel fish (5 g in 3 pieces of tail muscle) obtained from Badouzih fish-market were defrosted prior to experimentation.

(7) Light/ shade. Light from 2 swan neck cold lamps was provided from above. Shade was created by covering the entire treatment side half of the experimental tank by black velvet.

(8) Male/ female. A single male or female was exposed in the treatment compartment to the opposite sex in the choice area.

(9) Group of 6: six crabs (3 males and 3 females) were exposed in the treatment compartment to either, a single male and a single female in the choice area.

(10) Air-bubbling was provided by a commercial air pump from regular silicon tube without air stone at mild bubbling of 5 L air per minute in the treatment compartment.

(11) Mix of factors I (HV condition): Sulfur sediment (5g), elevated temperature, air-bubbling, shade were provided on the treatment side.

(12) Mix of factors II (non-HV condition): Quartz sediment, low temperature, no air-bubbling, light was provided on the treatment side.

Sixteen individual crabs of balanced gender ratio were tested individually between 9.00 and 18.00 hours of the day within 48 hours after capture. The position of the treatment and control side of the two-choice apparatus was reversed to avoid position bias after every five batches of crabs had been tested. The setup was cleaned after 10 batches had been tested. Each crab spent 5 min. in the two-choice chamber. A "no choice" was recorded for the rare case when a crab remained inactive for the whole experimental duration. A crab was considered to have made a ‘first choice’ when it moved >5 cm to either side (visually assessed by a line marked in the middle of the container). The ‘final choice’ of crab was the side they chose at the end of a 5-min experimental period. In addition was the amount of time crabs spent on each side of the device recorded. The experimental protocol followed otherwise the criteria provided by Train [37].

### Statistical analysis

Statistical testing for crab larvae distribution and crab habitat choice behavior were using SPSS version 13.0 for Windows software package (SPSS, Chicago, IL, U.S.A.). For the analyses of spatial distribution of crab larvae, one-way analysis of variance (ANOVA) with post-hoc Tukey’s honestly test was applied for abundance check among three depth samples following Zar [38]. A Chi-Square Test was used to determine the significance of differences between the number of crabs choosing the treatment or control side of the binary-choice experimental container. Crabs that did not make a choice as defined above were excluded from the statistical analyses.

## Results

### Distribution of *X*. *testudinatus* larvae at different depths

**Fig 3:**
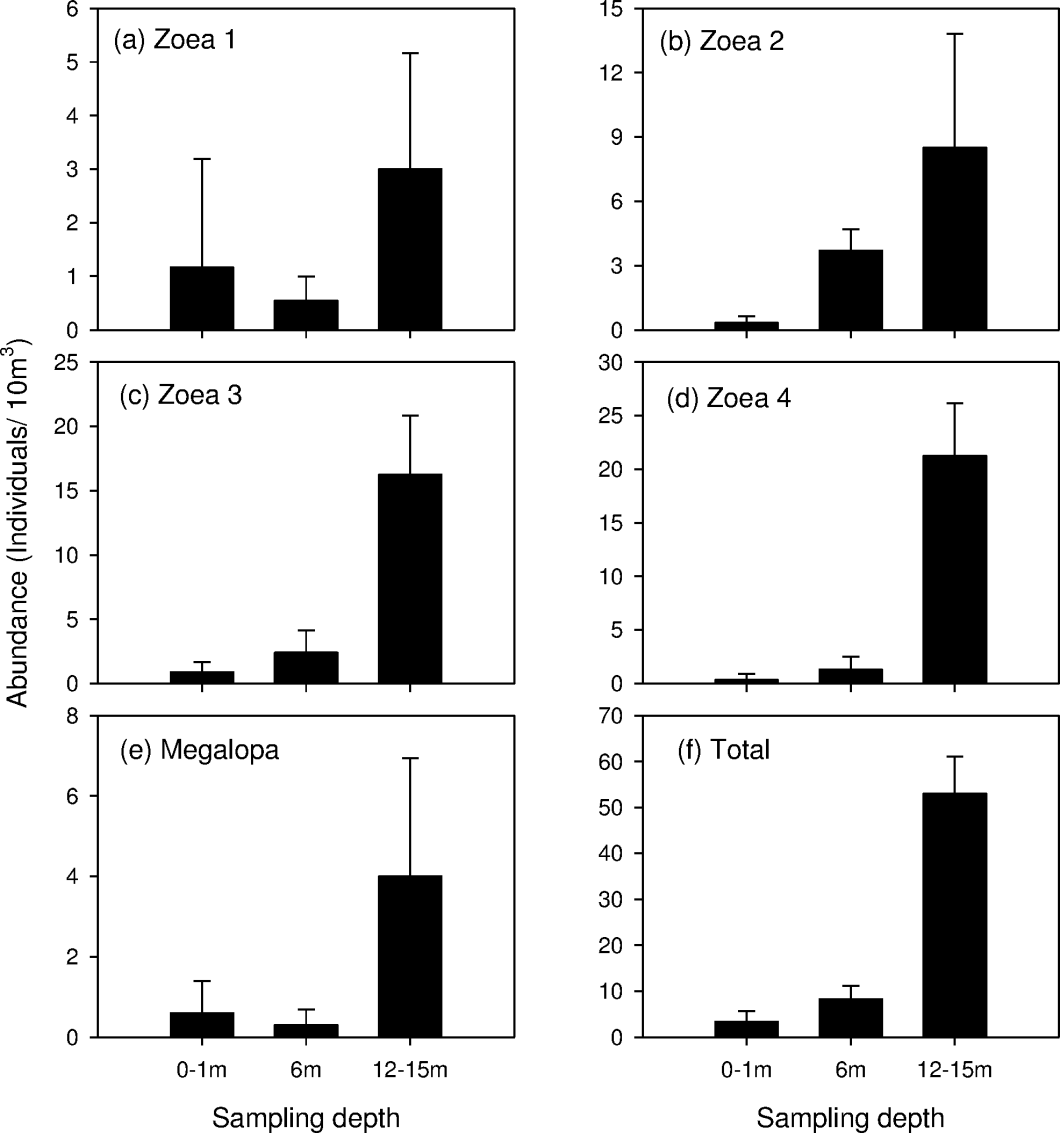
Average crab larval numbers at three depths: 1, 6, 12-15m.

Indications are provided from laboratory observations of the behavior of pelagic larval zoea and benthic megalopa stages of *X*. *testudinatus* that they tend to stay near the bottom even when swimming. The field distribution of larvae clearly indicates that its 4 zoea and 1 megalopa stages are distributed throughout the water column (Fig 3). Their abundances increase, however, substantially with depth showing highest densities at the sea-bottom.

Crab larvae zoea 1, zoea 2, zoea 3, zoea 4, and megalopa were identified and counted from samples at three depth strata. The abundance of larvae was commonly low in surface waters compared to deeper waters. The densities of crab larvae were on average 3.5 (individuals/10m^3^). The abundance of five stages larvae were not significantly different in the surface water (Fig 3A) although zoea 1 showed a relatively higher proportion in surface waters than the other stages. Crab larvae zoea 2, zoea 3 and zoea 4 were dominant in the bottom water layer (Fig 3B). In the water layer close to the seabed (12–15 m), the density of crab larvae increased. Overall was the Zoea 4 larval stage dominant and the highest recorded abundance was 27 (individuals/10m^3^). All zoea and megalopa crab larvae were higher near the bottom layer than in samples collected from the surface and from 6 m depth (Fig 3A–3C).

**Fig 4:**
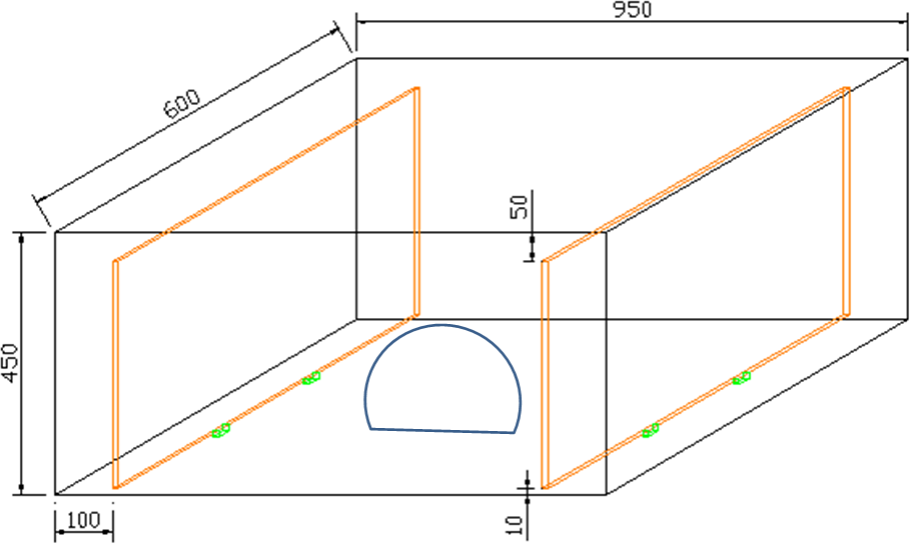
Experimental set-up of the binary choice experiment in the laboratory (metric unit: mm).

The statistical analysis of one-way ANOVA demonstrated different patterns of crab larvae distribution at three depths (Fig 4). The densities of zoea 1 larvae in samples of three depths were not different (*p* > 0.05) (Fig 4A). Zoea 2 larvae densities were significantly higher in deeper zone than surface zone (*p* = 0.029). In particular were densities of zoea 3 and zoea 4 larvae significantly higher in samples of 12-15m depth than at the surface, 0-1m (*p* < 0.001) and at 6m (*p* < 0.001) (Fig 4C and 4D). Megalopa larvae show higher densities in 12-15m samples but without significant difference among three depths (Fig 4E). Finally, we tested the abundance of total crab larvae at three depths. The results clearly indicated that deeper water samples contained significantly more crab larvae than the surface zone (*p* < 0.001) and middle zone (*p* < 0.001) (Fig 4F). Previous results showed a trend for crab larvae to be distributed in deeper layers with stage development.

### Habitat selection of crab responses to cues tested in a binary-choice tank

**Fig 5:**
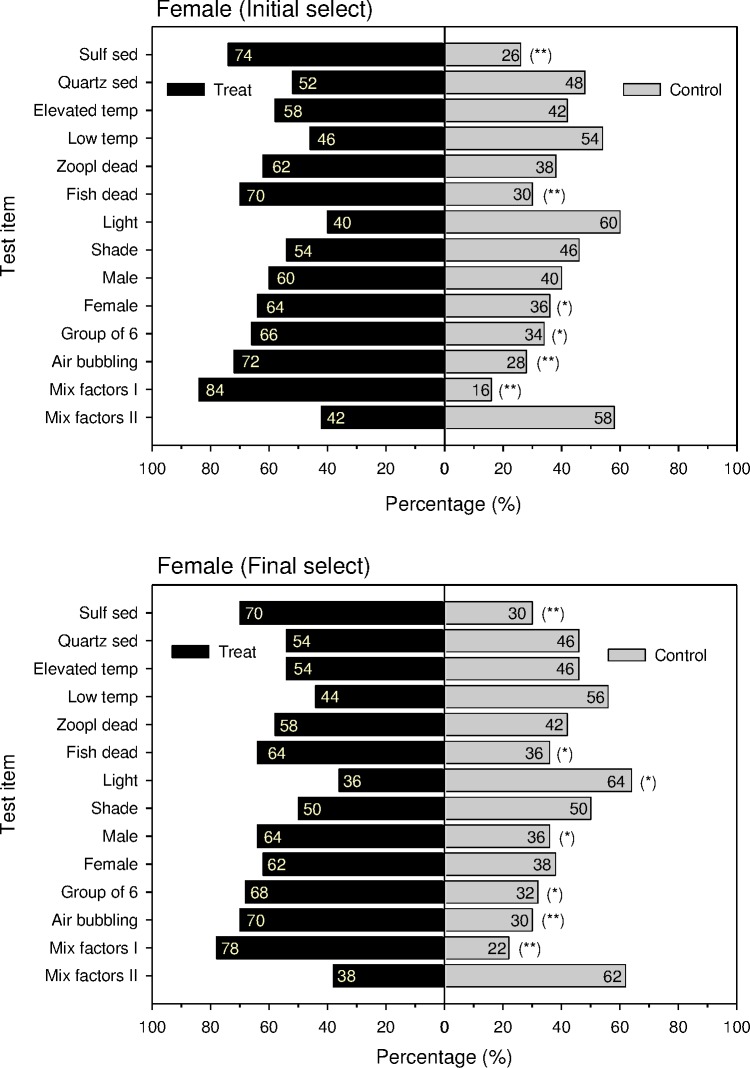
Binary choice of naive female *Xenograpsus testudinatus* in a binary-choice test. **Response of initial selection and final selection.** Numbers represent the percentage of selection behavior. * is *p* < 0.05; ** is *p* < 0.01; *** is *p* < 0.001.

**Fig 6:**
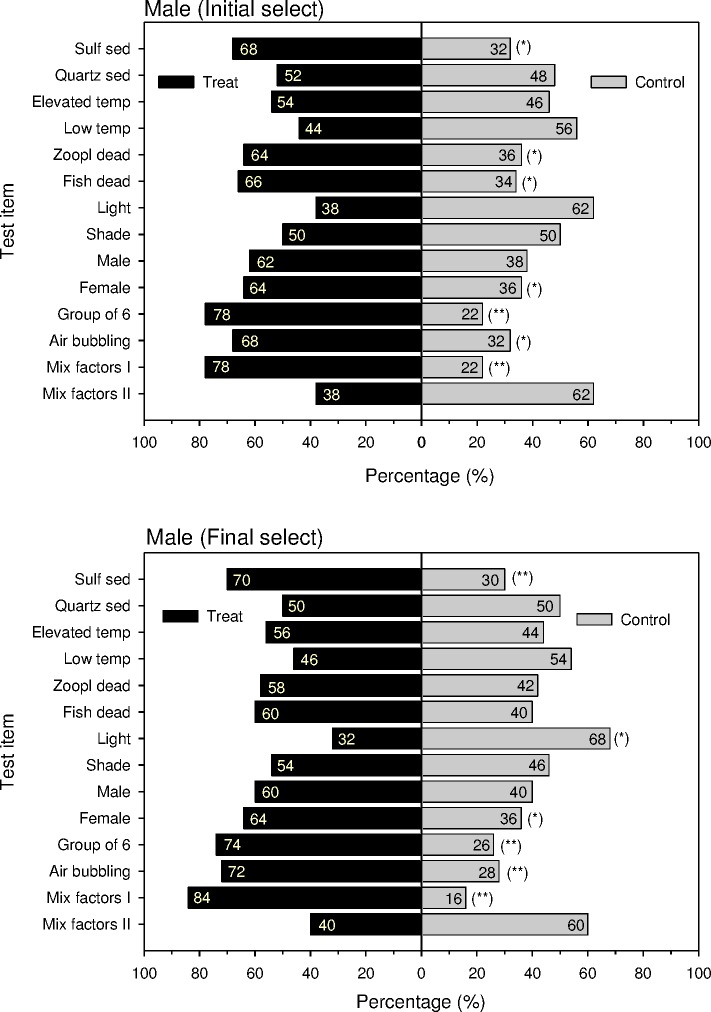
Binary choice of naive male *Xenograpsus testudinatus* in a binary-choice test tank. **Response of initial selection and final selection.** Numbers represent the percentage of selection behavior. * is *p* < 0.05; ** is *p* < 0.01; *** is *p* < 0.001.

Vent crabs that were collected at the HV-outlet of a shallow water hydrothermal vent site off Turtle Island and released about 10m away from the gas bubble- and flume-emitting outlet, they straightly swim-crawled back in the direction of the outlet. This way they showed their attraction to hitherto unknown HV site attractants. In our behavioral experiments in the lab as their first choice, naive females of *X*. *testudinatus* always significantly preferred vent-associated cues (percent responses > 70% in all cases, *p* < 0.01; Fig 5). The results of habitat selection (Table 1) varied between female crabs (Fig 5) and male crabs (Fig 6). When compared with expected distributions, crabs, irrespective of gender, significantly avoided light and tended to select other crabs, air-bubbling, sulfuric sediment, elevated temperature, dead fish, dead zooplankton, and quartz sediments in the order of decreasing importance, when the cues were offered alone and no such cue as a control in a two-choice setup. Sulfuric sediments and dead fish were significantly more attractive to females, and other crabs were significantly more attractive to males. Data do not support the hypothesis that dead settled zooplankton as a potential food source nor the other gender was significantly preferred. A combination of several vent-associated cues (sulfuric sediment, elevated temperature, air-bubbling) facilitated the strongest attraction to the crabs as shown by all response variables.

Despite the observed differences in crabs responding to the seven cues between the first choice and the final choice, the time they spent in the compartment containing each of the seven compounds was significantly longer than that in the control compartment (percent time > 61% in each case, *p* < 0.01). Furthermore, a combination of the HV-associated cues (elevated temperature, air-bubbling, sulfuric sediment) was significantly more attractive (80% response as first choice, *p* < 0.01; 74% response as a final choice, *p* < 0.01; and 69.1% residence time in the treatment compartment, *p* < 0.001).

## Discussion

Only a few organisms can survive at HVs. *Xenograpsus testudinatus*, discovered and identified as a new species in 2000 [17], is the only metazoan and macro- crustacean that can survive close to the outlets of HV effluents. The effluents in this environment are low-pH, low-temperature, high-sulfide, and metal-enriched. However, very little is known about the biological and chemical characteristics of this crab species (Hwang et al., 2008). As reported by Hwang et al. [2] do HV chimneys provide structurally complex three-dimensional habitats for larval and juvenile crabs of *X*. *testudinatus*. Jeng et al. [39] found that zooplankton being deadly affected by the vent plumes caused the vent crab Xenograpsus testudinatus to feed on dead zooplankton. Hwang et al. [2] found megalopa stages and juvenile crabs in the fissures and crevices of sulfur aggregates. These microhabitats have cavities that at least in part have been created by the juvenile crabs themselves [2]. Settlement in habitats with adequate protection, food, and temperature, would be critical for the survival of individuals at early crab stages. Settlement on unprotected bottoms would leave young crabs exposed to both predation, and dislodgement by currents. The spatial heterogeneity of sulfur aggregates may hence provide critical habitat properties for settlement and a nursery refuge for early juvenile crabs.

### Supply side ecology

Factors affecting larval input to the benthos might be especially important in structuring both populations and communities, and even supercede the importance of post-settlement processes such as competition, predation and physical disturbance. Amongst hydrothermal vent assemblages, larval supply processes do appear especially important since they are generally fairly isolated and ephemer at longer time scales. No comprehensive evaluation of the relative importance of colonization and recruitment has ever been undertaken at HVs. This may largely be due to the difficulty in obtaining samples or make experiments in such commonly difficult to access commonly deep sea environment. Shallow HVs provide a great advantage here since sampling and experimentation can be carried out by SCUBA diving [40].

Among larval ecologists, consensus is that hydrodynamic factors, density dependent effects of food availability and post-settlement processes are of prime importance [41].

How crabs become dispersed and recolonize the fluctuating habitats of newly developed HV sites or how they persist in their generally isolated habitats, is unknown as yet. It is unknown whether vent crabs show other than the daily migratory behavior towards food patches [42]. Low crab abundances during winter at vent sites of Turtle Island may suggest that individuals move into deeper waters and move back to shallower sites during summer. Reproduction is only interrupted for a winter break of about 4 months (author’s unpubl. data). In laboratory studies, we successfully reared the megalopa larvae through metamorphosis and several juvenile stages [2]. Our behavioral studies with both, zoea and megalopa stages showed that these larvae swim actively over the range of temperatures near the vents (12 to 32°C).

Dispersal and recruitment at HV sites remain generally hot topics for the supply side ecology in general [43–45] and at hydrothermal vents in particular [46]. Sufficient dispersal ability is important since HVs commonly are of short persistence and HV organisms need to frequently translocate to newly formed HV sites [1]. On the other hand are HV sites comparatively sparsely distributed and HV organisms are expected to have evolved strategies to stay at the spot not to get lost in the vast, likely unsuitable surroundings. Although the main dispersal stages might be the pelagic larvae, crab adults have good locomotory swim-crawling abilities and may also have evolved recognition systems to vent signaling cues as hypothesized and shown here. The attractive cues, however, remained elusive, since experiments are difficult to carry out in the commonly deep water sites of HVs. It has particularly not been demonstrated for invertebrates from neither shallow but commonly deep HVs as yet whether they can use various environmental cues to locate their vent habitat [46]. The HVs at Turtle Island provide a unique opportunity for an experimental approach to the supply side ecology of vent fauna in shallow waters.

#### - Habitat cues

Jeng and co-workers [39] found an enormously high density of crabs, *X*. *testudinatus*, living at the hydrothermal vent area off Turtle Island. The authors found that these crabs swarm out of their crevices during slack water to feed on the vast numbers of zooplankton that are killed by the vent’s sulphurous plumes. The zooplankton is so abundant that it rains down like marine ‘snow’.

The sediments at the HVs of Turtle Island are otherwise sulfuric [24] in contrast to the common quartz sediments in the marine environment. The sulfur sediments are densely colonized by [47]. Micoorganisms which may provide an additional biofilm cue to the factor sediment [48]. The gasing or bubbling of different HV gases is a constitutive charactersistic at vents side and also clearly prefered in our dual choice experiments [49, 50]. Elevated temperatures provide another vent specific cue Macrobiota from HVs were shown to have distinct receptory capabilities. For the detection of temperature differences and show selection for temperature preferenda [8, 9].

#### - Experimental approach

A frequent criticism of many binary-choice experiments is to ignore that experimental animals make mistakes during initial explorations of a binary-choice set-up. Du and co-workers [51] suggested, therefore, that recording the animals' final choices after a set time period would solve this problem. Since the crabs spent longer time on the side of the binary-choice device that contained the preferred cue, we also measured the time crabs spent on each side of the binary-choice tank. Our data show that the first choice responses of naive female *X*. *testudinatus* were always consistent with the side of the tank where they spent the greatest amount of time but not with the final choice of the crabs (Figs 5 and 6). For instance, irrespective of differences in their first and final choices, crabs tended to spend a greater amount of time exploring the side of the tank that contained any of the cue components than that containing the control (Figs 5 and 6). This suggests that recording the time that crabs spend at each side of the binary-choice tank, in addition to recording their final choice, is more reliable than just recording their final choice. Recording only the first choice of the crabs was sufficient and would have greatly reduced our experimental time efford.

## Perspectives

As shown here and in previous studies does the marine environment adjacent to Turtle Island provides a natural laboratory for different studies, such as for ecotoxicology, behavior, comparative physiological and molecular studies, biotechnology applying the genetic diversity in an applied technological context. Particular climate change studies can make use of related environmental factors in this natural laboratory. Since key marine environments such as shallow water HVs are highly threatened by various anthropogenic usages and disturbances does their protection become a critical issue. Marine environ-mental protection became a most urgent issues worldwide and marine protected areas (MPAs) became established. Taiwan also plans to increase the size of Taiwan's MPAs to 20% of its total ocean territory within this decade and Kueishan Island has to be one of the focus points of protection also to ensure the sustainability of a future ocean with all its diverse ocean landscapes and biotic adaptations from molecular to ecosystem scale.

## References

[1] Van Dover C. The ecology of deep-sea hydrothermal vents Princeton University Press; 2000.

[2] Hwang J-S, Dahms H-U, Alekseev V. Novel nursery habitat of hydrothermal vent crabs. Crustaceana. 2008; 81: 375–380. doi: 10.1163/156854008783564127

[3] Sarradin PM, Caprais JC, Briand P, Gaill F, Shillito B, Desbruyères D. Chemical and thermal description of the environment of the Genesis hydrothermal vent community (13°N, EPR). Cah Biol 3 1998; 39:159–167.

[4] Colaco A, Bustamante P, Fouquet Y, Sarradin PM, Serrão-Santos R. Bioaccumulation of Hg, Cu, and Zn in the Azores triple junction hydrothermal vent fields food web. Chemosphere. 2006; 65: 2260–2267. doi: 10.1016/j.chemosphere.2006.05.034 16844198 10.1016/j.chemosphere.2006.05.034

[5] Haraguchi K, Ando T, Sato M, Kawaguchi C, Tomiyasu T, Horvat M, Akagi H. Detection of localized methylmecury contamination by use of the mussel adductor muscle in Minamata Bay and Kagoshima Bay, Japan. Sci Tot Environ. 2000; 261: 75–89. 10.1016/s0048-9697(00)00626-4 11036979

[6] Chen CTA, Zeng Z, Kuo FW, Yang TF, Wang BJ. Tide-influenced acidic hydrothermal system offshore NE Taiwan. 2005 Chem. Geol. 224, 69–81.

[7] Hsiao S-H, Fang T-H. Hg bioaccumulation in marine copepods around hydrothermal vents and the adjacent marine environment in northeastern Taiwan. Mar Poll Bull. 2013; 74:175–182. 10.1016/j.marpolbul.2013.07.007 23932475

[8] Childress JJ, Fisher CR. The biology of hydrothermal vent animals: physiology, biochemistry, and autotrophic symbioses. Oceanogr Mar Biol. 1992;30:337–441.

[9] Van Dover CL, German CR, Speer KG, Parson LM, Vrijenhoek RC. Evolution and biogeography of deep-sea vent and seep invertebrates. Science. 2002; 295: 1253–1257. doi: 10.1126/science.1067361 11847331 10.1126/science.1067361

[10] Fisher CR. Chemoautotrophic and methanotrophic symbioses in marine invertebrates. Rev Aquat Sci. 1990;2:399–436. doi: 10.1111/j.1439-0485.1993.tb00001.x

[11] Cosson RP, Vivier JP. Interactions of metallic elements and organisms within hydrothermal vents. Cah Biol 3 1997; 38: 43–50.

[12] Desbruyeres DM, Segonzac M, Bright M. Hydrothermal vent animals–Identification Linz Museum 2006; 1–544.

[13] Jinks RN, Battelle BA, Herzog ED, Kass L, Renninger GH, Chamberlain SC. Sensory adaptations in hydrothermal vent shrimps from the Mid-Atlantic Ridge. Cah Biol 3 1998; 39:309–312.

[14] Chen CTA, Wang BY, Huang JF, Lou JY, Kuo FW, Tu YY, et al Investigation into extremely acidic hydrothermal fluids off Kueishan Tao, Taiwan, China. Acta Oceanol Sin. 2005; 24:125–133.

[15] Dahms H-U, Tseng L-C, Shim DM-C, Hwang J-S. Hydrothermal Vent Effluents Affect Life Stages of the Copepod *Tisbe* sp. J Mar Sci Technol. Taiwan Ocean University; 2014; 22:82–88.

[16] Lu S-Y, Shen C-H, Chiau W-Y. Zoning strategies for marine protected areas in Taiwan: Case study of Gueishan Island in Yilan County, Taiwan. Marine Policy. 2014; 48: 21–29.

[17] NG N. K., HUANG J. F., AND HO P. H. Description of a new species of hydrothermal crab, *Xenograpsus testudinatus* (Crustacea: Decapoda: Brachyura: Grapsidae) from Taiwan. Natn. Taiwan. Mus. Spec. Publ. Ser. 2000; 10:191–199.

[18] Hwang J-S, Wang J, Chen T. Proceedings of the International Symposium on Marine Biology in Taiwan—Crustacean and Zooplankton Taxonomy, Ecology and Living Resources. International Symposium on Marine Biology in Taiwan-Crustacean and Zooplankton Taxonomy, Ecology and Living Resources (1998: National Taiwan Ocean University, Keelung, Taiwan). National Taiwan Museum; 2000; 10:1–199.

[19] Hwang JS, Lee CS. The mystery of underwater world for tourism of Turtle Island, Taiwan. Northeast Coast Natl Scen Area Adm Tour Bur Minist Transp Commun Taiwan (in Chinese). 2003; 1–103.

[20] Chengsung Wang M-LY, Chou C-P, Chang Y-C, Lee C-S. Westward Extension of the Okinawa Trough at its Western End in the Northern Taiwan Area: Bathymétrie and Seismological Evidence. TAO Terr Atmos Ocean Sci. Meteorological Society, Geological Society and Geophysical Society of the ROC; 2000; 11:459–480.

[21] Wang L, Cheung MK, Kwan HS, Hwang J, Wong CK. Microbial diversity in shallow‐water hydrothermal sediments of Kueishan Island, Taiwan as revealed by pyrosequencing. J Basic Microbiol. Wiley Online Library; 2015; 55:1308–1318. doi: 10.1002/jobm.201400811 26132902 10.1002/jobm.201400811

[22] Robigou V, Delaney JR, Stakes DS. Large massive sulfide deposits in a newly discovered active hydrothermal system, the High‐Rise field, Endeavour segment, Juan de Fuca Ridge. Geophys Res Lett. Wiley Online Library; 1993; 20:1887–1890.

[23] Yang TF, Lan TF, Lee HF, Fu CC, Chuang PC, Lo CH, et al Gas compositions and helium isotopic ratios of fluid samples around Kueishantao, NE offshore Taiwan and its tectonic implications. Geochem J. 2005; 39:469–480. doi: 10.2343/geochemj.39.469

[24] Chan BKK, Wang T-W, Chen PC, Lin C-W, Chan TY, Tsang LM. Community Structure of Macrobiota and Environmental Parameters in Shallow Water Hydrothermal Vents off Kueishan Island, Taiwan. PLoS ONE. 2016; 11(2): e0148675 doi: 10.1371/journal.pone.0148675 26849440 10.1371/journal.pone.0148675 PMC4744018

[25] McLay C. New crabs from hydrothermal vents of the Kermadec Ridge submarine volcanoes, New Zealand: *Gandalfus* gen. nov. (Bythograeidae) and *Xenograpsus* (Varunidae) (Decapoda: Brachyura). Zootaxa. 2007; 1–22.

[26] Martin JW, Haney TA. Decapod crustaceans from hydrothermal vents and cold seeps: A review through 2005. Zoological Journal of the Linnean Society. 2005 pp. 445–522. doi: 10.1111/j.1096-3642.2005.00178.x

[27] Williams AB. A new crab family from the vicinity of submarine thermal vents on the Galapagos Rift (Crustacea: Decapoda: Brachyura). Proc Biol Soc Washingt. 1980; 93: 443–472.

[28] Ki JS, Dahms HU, Hwang JS, Lee JS. The complete mitogenome of the hydrothermal vent crab *Xenograpsus testudinatus* (Decapoda, Brachyura) and comparison with brachyuran crabs. Comp Biochem Physiol—Part D Genomics Proteomics. 2009; 4: 290–299. doi: 10.1016/j.cbd.2009.07.002 20403751 10.1016/j.cbd.2009.07.002

[29] Tunnicliffe V, McArthur AG, McHugh D. A Biogeographical Perspective of the Deep-Sea Hydrothermal Vent Fauna [Internet]. Advances in Marine Biology. 1998 doi: 10.1016/S0065-2881(08)60213-8

[30] Hessler RR, Wilson GDF. The origin and biogeography of malacostracan crustaceans in the deep sea. Evol time Sp Emerg Biosph. Academic Press, London; 1983; 227–254.

[31] Newman WA. The abyssal hydrothermal vent invertebrate fauna: A glimpse of antiquity? Bull Biol Soc Washingt. 1985; 6: 231–242.

[32] Van Dover CL, Factor JR, Williams AB, Berg CJ Jr. Reproductive patterns of decapod crustaceans from hydrothermal vents. Bull Biol Soc Washingt. 1985; 223–227. doi: 10.2307/1380793

[33] Guinot D, Segonzac M. Description d’un crabe hydrothermal nouveau du genre *Bythograea* (Crustacea, Decapoda, Brachyura) et remarques sur les Bythograeidae de la dorsale du Pacifique oriental. Zoosystema. Editions scientifiques du Muséum; 1997; 19:121–149.

[34] Epifanio CE, Perovich G, Dittel AI, Cary SC. Development and behavior of megalopa larvae and juveniles of the hydrothermal vent crab *Bythograea thermydron*. Mar Ecol Prog Ser. 1999; 185: 147–154. doi: 10.3354/meps185147

[35] Bauer RT. Chemical communication in decapod shrimps: The influence of mating and social systems on the relative importance of olfactory and contact pheromones. Chemical Communication in Crustaceans. 2011pp.277–296. doi: 10.1007/978-0-387-77101-4_14

[36] Dahms HU, Gao QF, Hwang JS. Optimized maintenance and larval production of the bryozoan *Bugula neritina* (Bugulidae: Gymnolaemata) in the laboratory. Aquaculture. 2007; 265: 169–175. doi: 10.1016/j.aquaculture.2007.01.029

[37] Train KE. Discrete Choice Methods with Simulation. Cambridge Univ Press 2003; 1–388. doi: 10.1017/CBO9780511753930

[38] Zar JH. Biostatistical analysis Pearson Education India; 1999.

[39] Jeng M-S, Ng NK, Ng PKL. Feeding behaviour: hydrothermal vent crabs feast on sea “snow”. Nature. 2004; 432: 969 doi: 10.1038/432969a 15616550 10.1038/432969a

[40] Todd CD. Recruitment, Colonization and Physical-Chemical Forcing in Marine Biological Systems Volume 132 of the series Developments in Hydrobiology. 1998; pp 1–21.

[41] Robins PE, Neill SP, Giménez L, Jenkins SR, Malham SK. Physical and biological controls on larval dispersal and connectivity in a highly energetic shelf sea. Limnol Oceanogr. 2013; 58:505–524

[42] Jeng M-S, Clark PF, Ng PKL. The first zoea, megalopa, and first crab stage of the hydrothermal vent crab *Xenograpsus testudinatus* (Decapoda: Brachyura: Grapsoidea) and the systematic implications for the Varunidae. J Crustac Biol. Brill; 2004; 24:188–212.

[43] Dahms HU, Qian PY. Exposure of biofilms to meiofaunal copepods affects the larval settlement of *Hydroides elegans* (Polychaeta). Mar Ecol Prog Ser. 2005; 297: 203–214. doi: 10.3354/meps297203

[44] Dahms HU, Harder T, Qian PY. Selective attraction and reproductive performance of a harpacticoid copepod in a response to biofilms. J Exp Mar Bio Ecol. 2007; 341: 228–238. doi: 10.1016/j.jembe.2006.10.027

[45] Dahms HU, Li X, Zhang G, Qian PY. Resting stages of *Tortanus forcipatus* (Crustacea, Calanoida) in sediments of Victoria Harbor, Hong Kong. Estuar Coast Shelf Sci. 2006; 67: 562–568. doi: 10.1016/j.ecss.2005.12.011

[46] Mullineaux LS, Mills SW, Sweetman AK, Beaudreau AH, Metaxas A, Hunt HL. Vertical, lateral and temporal structure in larval distributions at hydrothermal vents. Mar Ecol Prog Ser. 2005; 293: 1–16. doi: 10.3354/meps293001

[47] Vignesh S, Muthukumar K, Dahms H-U, Vignesh G, James RA. Multiple marker analysis of pollutants along the tropical coastal zone of southern India. PlosONE. (in print on 21 12 2016) 0154105.

[48] Wang L, Cheung MK, Kwan HS, Hwang J-S, Wong CK. Microbial diversity in shallow-water hydrothermal sediments of Kueishan Island, Taiwan as revealed by pyrosequencing. J Basic Microbiol. 2015; 55: 1308–1318. doi: 10.1002/jobm.201400811 26132902 10.1002/jobm.201400811

[49] German C. Oceanography: Bubbling under. Nature. 2002; 415: 124–125. doi: 10.1038/415124a 11805814 10.1038/415124a

[50] Bischoff JL, Rosenbauer RJ. Liquid-vapor relations in the critical region of the system NaCl H2O from 380 to 415°C: A refined determination of the critical point and two-phase boundary of seawater. Geochimica et Cosmochimica Acta. 1988; 52 (8): 2121–2126.

[51] Du YJ, Poppy GM, Powell W, Pickett J a, Wadhams LJ, Woodcock CM. Identification of semiochemicals released during aphid feeding that attract parasitoid *Aphidius ervi*. J Chem Ecol. 1998; 24:1355–1368. doi: 10.1023/A:1021278816970

